# Novel Carboxymethyl Cellulose-Based Hydrogel with Core–Shell Fe_3_O_4_@SiO_2_ Nanoparticles for Quercetin Delivery

**DOI:** 10.3390/ma15248711

**Published:** 2022-12-07

**Authors:** Mohammad Mahdi Eshaghi, Mehrab Pourmadadi, Abbas Rahdar, Ana M. Díez-Pascual

**Affiliations:** 1 Department of Biotechnology, School of Chemical Engineering, College of Engineering, University of Tehran, Tehran 1417935840, Iran; 2 Department of Physics, Faculty of Sciences, University of Zabol, Zabol 538-98615, Iran; 3 Universidad de Alcalá, Facultad de Ciencias, Departamento de Química Analítica, Química Física e Ingeniería Química, Ctra. Madrid-Barcelona, Km. 33.6, 28805 Alcalá de Henares, Madrid, Spain

**Keywords:** carboxymethyl cellulose, quercetin, Fe_3_O_4_ nanoparticles, core–shell nanoparticles, double-emulsion system

## Abstract

A nanocomposite composed of carboxymethyl cellulose (CMC) and core–shell nanoparticles of Fe_3_O_4_@SiO_2_ was prepared as a pH-responsive nanocarrier for quercetin (QC) delivery. The nanoparticles were further entrapped in a water-in-oil-in-water emulsion system for a sustained release profile. The CMC/Fe_3_O_4_@SiO_2_/QC nanoparticles were characterized using dynamic light scattering (DLS), Fourier transform infrared spectroscopy (FTIR), X-ray diffraction (XRD), a field emission scanning electron microscope (FE-SEM), and a vibrating sample magnetometer (VSM) to obtain insights into their size, stability, functional groups/chemical bonds, crystalline structure, morphology, and magnetic properties, respectively. The entrapment and loading efficiency were slightly improved after the incorporation of Fe_3_O_4_@SiO_2_ NPs within the hydrogel network. The dialysis method was applied for drug release studies. It was found that the amount of QC released increased with the decrease in pH from 7.4 to 5.4, while the sustained-release pattern was preserved. The A549 cell line was chosen to assess the anticancer activity of the CMC/Fe_3_O_4_@SiO_2_/QC nanoemulsion and its components for lung cancer treatment via an MTT assay. The L929 cell line was used in the MTT assay to determine the possible side effects of the nanoemulsion. Moreover, a flow cytometry test was performed to measure the level of apoptosis and necrosis. Based on the obtained results, CMC/Fe_3_O_4_@SiO_2_ can be regarded as a novel promising system for cancer therapy.

## 1. Introduction

Cancer is among the most commonly occurring diseases worldwide. In the United States, cancer is identified as the second most frequent cause of death. Statistics indicate that lung cancer accounts for largest share of cancer-related deaths in both men and women in the United States [1]. Even though chemotherapy is the prominent approach for curing various types of cancer, including lung cancer, its efficacy is limited due to factors such as drug resistance, non-specific targeting, severe side effects on healthy cells, and low bioavailability [2]. The mentioned drawbacks of chemotherapy indicate the need to use of less toxic drugs in conjunction with smart drug delivery systems, which can lead to less side effects and enhanced therapeutic effects [3].

Plant-based polyphenols have been extensively studied for their potential usage in cancer treatment. Among these natural compounds, quercetin (QC) has been recognized as a promising member in terms of antineoplastic activity. QC belongs to the flavonol family, which is a class of flavonoids. Studies have proved that quercetin has anti-oxidative, anti-inflammatory, and anticancer activity. Its anticancer activity is shown via different mechanisms, including metastasis inhibition, apoptosis induction, the disturbing of cell proliferation, angiogenesis disruption, oxidative stress suppression, and the influencing of autophagy [4,5,6,7,8,9]. Regarding the anti-tumor activity of QC against lung cancer tumors, studies have indicated that quercetin shows anti-proliferation and anti-metastasis behavior towards the A549 cell line by affecting the cytoskeleton of cancerous cells. The anti-proliferative behavior of quercetin towards A549 cells has been associated with a disturbance in cytokinesis during mitosis as a result of cytoskeleton components getting eliminated from the cytoplasm by quercetin [4,10]. It has been shown that QC can also improve radio-sensitivity of non-small cell lung cancer [11]. Further studies have proven that despite the complicated correlation between autophagy and apoptosis in different cancerous cell lines, autophagy inhibition can limit quercetin-induced apoptosis on the A549 cell line. Hence, in order for quercetin to be an effective therapeutic agent for lung cancer, it needs to be used in parallel with an initiator of autophagy [12].

The therapeutic effects of QC and similar flavonoid compounds have been limited by drawbacks such as low bioavailability [13], instability [14], poor solubility [15], and inefficient biodistribution [16]. To overcome these limitations, novel drug delivery platforms for enhanced therapeutic efficiency need to be developed. QC-loaded nanoparticles offer several advantages, such as long circulation time, controlled release, improved entrapment efficiency, and stability. Among different NPs, polymeric ones possess advantageous characteristics for drug delivery. They are mostly non-toxic, biodegradable, biocompatible, and stable [17,18,19]. About a hundred years ago, carboxymethyl cellulose (CMC) was manufactured in Germany for the first time. It is a highly water-soluble biological macromolecule. Apart from the abovementioned properties of polymeric NPs, CMC alleviates the side effects of drugs, enhances bioavailability, and improves anti-tumor activity. Furthermore, CMC can change its state from acid to base and vice versa depending on the pH. Hence, CMC is a pH-responsive polymer and a potentially smart nanocarrier. Since the extracellular environment of tumors has a lower pH than ordinary cells, the pH sensitivity of CMC can improve the targeted delivery of quercetin [20,21,22,23]. Polymeric compounds such as CMC can be cross-linked to form a hydrophilic network called hydrogel. Hydrogels can absorb large amounts of water without losing structural integrity. The porosity of these networks can be modified by controlling the cross-linking density. The large amount of water that can be absorbed by hydrogels, together with their stimulus-responsive properties, makes them suitable drug carriers. Owing to their pH and temperature sensitivity, their cargo can be released in a controlled way. In addition, their large water content makes them very biocompatible [24,25].

Despite the abovementioned advantages, hydrogels suffer from some drawbacks for usage as drug carriers. Due to their hydrophilic properties, encapsulating hydrophobic drugs in them can be challenging. Stimulus-responsive hydrogels demonstrate very low response time, and they are not homogeneous in general. Furthermore, they have low mechanical strength and a high rate of biodegradability [3,26,27,28,29,30,31]. One solution to circumvent the mentioned disadvantages is to incorporate inorganic nanoparticles within the hydrogel structure, developing nanocomposites. Introducing inorganic NPs can improve the mechanical properties of hydrogels, increase drug loading capacity, make them stimulus responsive, and enhance biocompatibility, thereby improving drug delivery efficacy [32]. Among inorganic nanoparticles, iron oxide-based ones and magnetite (Fe_3_O_4_) in particular have received considerable attention for drug delivery applications. Fe_3_O_4_ demonstrates outstanding magnetic and electronic properties and is sufficiently biocompatible [33,34]. Previous research conducted by Azizi [33] has proven that introducing magnetite NPs within a polymeric structure can enhance the swelling capacity, loading capacity, and thermal resistance of the nanocarriers. These improvements can be associated with the electrical properties and high surface area of magnetite. In another study, Mohammadi et al. [35] fabricated carboxymethylcellulose/polyacrylic acid/starch-modified Fe_3_O_4_ nanocomposite hydrogels for oral doxorubicin delivery. The swelling ratio of nanocomposites depended on both pH and the weight percentage of Fe_3_O_4_. This contributed to controlled drug release in the intestine (neutral environment) instead of the stomach (acidic environment). However, high surface area and instability at low pH can lead to the aggregation and oxidation of Fe_3_O_4_ molecules. Other inorganic nanoparticles with suitable characteristics for drug delivery are SiO_2_. These NPs have high surface area and an amphiphilic surface and are biocompatible. Furthermore, various functional groups can be added to their surface thanks to the valence electrons of silicon atoms. Hence, they can be used for delivering cargos such as antineoplastic drugs [36,37]. In order to make up for the drawbacks of Fe_3_O_4_, core–shell NPs of Fe_3_O_4_ coated with SiO_2_ can be used. Core–shell NPs have several advantages over normal NPs, including enhanced biodistribution, lower cytotoxicity, more controlled release of the drug, improved binding to biomolecules, and stimulus-responsive properties [38,39].

**Scheme 1:**
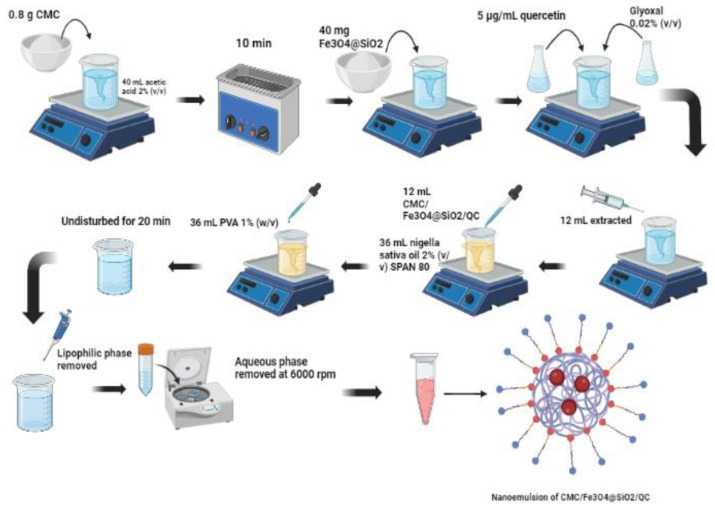
Schematic representation of the procedure for the synthesis of double-nanoemulsion-entrapped nanocomposite of CMC/Fe_3_O_4_@SiO_2_/QC.

Herein, we prepared a hydrogel nanocomposite based on CMC that incorporated Fe_3_O_4_@SiO_2_ core–shell nanoparticles (Scheme 1). QC was then loaded in the CMC/Fe_3_O_4_@SiO_2_/QC nanocomposite. Since hydrogels have aqueous environments, loading hydrophobic drugs such as quercetin inside them can be challenging. However, the presence of core–shell NPs within the hydrogel structure provided a large surface area and made a slight contribution to improving the loading capacity of quercetin. The majority of the loaded quercetin was localized in the porous structure of SiO_2_ (shell material). There have been reports of possible hydrogen bonding between CMC and quercetin, with the hydroxyl group of quercetin as the donor, in the literature. These interactions lead to the formation of a stable network comprising CMC and quercetin [40,41]. In addition, there have been reports of using Fe_3_O_4_@ SiO_2_ core–shell nanoparticles for adsorbing quercetin and other flavonoids [42,43]. These reports justify our choice of material for fabricating the drug delivery system. Furthermore, we examined the pH sensitivity of the prepared delivery system by analyzing the release behavior at two different pH values (5.4 and 7.4). The obtained results demonstrated a sustained but improved release of quercetin at the lowest pH. The sensitivity of CMC/Fe_3_O_4_@SiO_2_/QC to pH could be associated with the protonation of carboxyl groups at low pH values, which leads to a reduction in electrostatic interactions between the drug and the carrier. These elucidations are in agreement with previous literature reports [44,45,46]. The preparation of hydrogel nanocomposites was followed by a water-in-oil-in-water (W-O-W) emulsification step. Loading the nanocomposites in W-O-W nanoemulsions has many advantages. Nanoemulsions provide benefits such as controlling the release of quercetin, inhibiting its degradation, biocompatibility, and capability for loading both hydrophilic and lipophilic drugs [47,48].

Therefore, the purpose of this research was to introduce a novel nanocarrier for treating cancer using QC. The pH sensitivity of the nanocarrier reduced the side effects of quercetin. In addition, the incorporation of core–shell nanoparticles slightly improved the drug loading in the polymeric carrier. Although the loading and entrapment efficiency did not improve significantly after introducing core–shell nanoparticles, the FTIR analysis revealed that in the presence of these nanoparticles, a share of loaded quercetin is entrapped inside them instead of the polymeric network. This entrapment contributes to the sustained release of quercetin, as the drug has to diffuse through extra layers before getting released. The effectiveness of the system was assessed against the A549 cell line. XRD and FTIR characterization was used to determine the crystalline structure, physical properties, and composition of the samples. A VSM test was employed to confirm the magnetic properties of Fe_3_O_4_. In addition, FESEM images were recorded to obtain insights into the morphology of the nanocarriers.

## 2. Materials and Methods

### 2.1. Materials

Sodium carboxymethyl cellulose (M_w_ = 90,000) was purchased from Merck Co. (Darmstadt, Germany). FeCl_3_.6H_2_O (reagent grade, 97%) was purchased from Sigma Aldrich Co. (Burlington, MA, USA). Quercetin drug (>95% (HPLC), solid) was also obtained from Sigma Aldrich Co. SPAN 80 (molar mass = 428.60 g/mol) was purchased from Merck Co. Phosphate-buffered saline was obtained from Sigma Aldrich Co. Ammonium hydroxide solution and tetraethyl orthosilicate (M_w_ = 208.33) were obtained from Merck Co. (Darmstadt, Germany).

### 2.2. Preparation of Fe_3_O_4_ Nanoparticles

In order to synthesize Fe_3_O_4_ nanoparticles, 4.8 g of FeCl_3_.6H_2_O was added to 100 mL of deionized water. The solution was stirred at 700 rpm for 1 h under Ar atmosphere conditions, and the salts were completely dissolved in the water. Then, 10 mL of ammonium hydroxide (25%) was added to the solution dropwise over 10 min. The black precipitate of magnetite nanoparticles was formed instantly. This was followed by 1 h of mechanical stirring. The precipitate was then removed using an external magnet and washed five times using distilled water. Finally, the NPs were dried at 50 °C overnight [49].

### 2.3. Preparation of Fe_3_O_4_@SiO_2_ Core–Shell Nanoparticles

The synthesis process began with dissolving 1 g of magnetite nanoparticles in a solution with 40 mL of ethanol and 10 mL of water using an ultrasonic bath. The solution was then transferred to a three-necked bottle. The pH of the solution was fixed at 10 using ammonia solution. A volume of 0.5 mL of tetraethyl orthosilicate was then added to the solution dropwise, and the resulting mixture was stirred for 6 h at 50 °C. Finally, core–shell nanoparticles of Fe_3_O_4_@SiO_2_ were formed. The NPs were then thoroughly washed with ethanol and distilled water and dried at 60 °C for 24 h [49].

### 2.4. Preparation of Quercetin-Loaded CMC/Fe_3_O_4_@SiO_2_ Hydrogel

The overall protocol for the preparation of the hydrogel was similar to those found in previous literature reports [50]. Firstly, 0.8 g of CMC was added to 40 mL of acetic acid 2% (*v*/*v*) solution. The solution was placed on a heater stirrer until complete dissolution of the polymer at room temperature was achieved and homogenous 2% (*w*/*v*) CMC solution was obtained. The homogenous solution was then placed in an ultrasonic bath for 10 min. Then, 40 mg of Fe_3_O_4_@SiO_2_ NPs was added to the mixture, and the solution was placed on a heater stirrer until homogeneity was achieved. Then, 0.02% (*v*/*v*) glyoxal as the crosslinking agent was added to the mixture. The quantity of QC added was chosen so that the final concentration of the drug in the hydrogel was 5 µg/mL. After QC addition, the mixture was heated while stirring for another 30 min; finally, CMC/Fe_3_O_4_@SiO_2_/QC hydrogel was obtained.

### 2.5. Preparation of Double-Emulsion-Encapsulated Hydrogel

The double-emulsion system was prepared using the method reported by Ahmadi et al. [51]. A volume of 12 mL of CMC/Fe_3_O_4_@SiO_2_/QC hydrogel was extracted using a syringe and added to 36 mL of 2% (*v*/*v*) SPAN 80-containing nigella sativa oil dropwise (SPAN 80 as the hydrophobic surfactant). The hydrophobic phase was placed on a stirrer during this operation. Upon the addition of the hydrogel to the hydrophobic phase, spherical particles of nanocarriers were formed in the solution. After 10 min of stirring, 36 mL of PVA 1% (*w*/*v*) solution (PVA as the hydrophilic surfactant) was added to the mixture dropwise. Then, the stirring process was stopped, and the system was kept undisturbed for 20 min so that different layers could become separated. The lipophilic phase was then separated by means of a sampler. The aqueous phase was centrifuged at 6000 rpm, and the water was removed from the QC-loaded nanocarriers.

Prior to each test, the materials were powdered using a freeze-dryer. The samples were subjected to a temperature of −20 °C prior to freeze-drying.

### 2.6. Characterization of Nanoparticles

The morphology of nanoparticles was observed using a field emission scanning electron microscope (FE-SEM). Dynamic light scattering (DLS) was performed to determine the size distribution and zeta potential of the nanoparticles. X-ray diffraction (XRD) was employed to analyze the crystalline structure of the nanocomposite after the addition of each component. Fourier transform infrared (FTIR) spectroscopy was used to identify the different functional groups in the composite and to assess the interactions between the different components in the nanocomposite network. Finally, a vibrating sample magnetometer was used to verify the magnetic property of Fe_3_O_4_.

### 2.7. Drug Loading and Encapsulation Efficiency

The method applied for measuring loading and encapsulation efficiency is similar to the approach reported in the literature [52]. In order to determine the effect of incorporating core–shell nanoparticles within the polymeric network on the loading capacity and entrapment efficiency, CMC/QC and CMC/Fe_3_O_4_@SiO_2_/QC NPs were added to phosphate-buffered saline. Next, ethyl acetate solvent was added to the mixture and stirred until homogeneity was obtained. The organic phase was then separated, and its quercetin content was measured using UV-Vis spectrophotometer. Equations (1) and (2) were used for calculating the loading efficiency and encapsulation efficiency, respectively.

(1) $$Loading\;Efficiency\;\left(\%\right)=\frac{\left(Total\;QC\;quantity\right)-\left(Free\;QC\;quantity\right)}{Total\;nanocarrier\;quantity}$$

(2) $$Entapment\;Efficiency\;\left(\%\right)=\frac{\left(Total\;QC\;quantity\right)-\left(Free\;QC\;quantity\right)}{Total\;QC\;quantity}$$

### 2.8. In Vitro Drug Release

The dialysis method was applied to study the release profile of the drug from nanoemulsions in vitro [53]. A beaker was filled with water at 37 °C to simulate the thermal conditions of the body. The beaker was placed on a magnetic stirrer to ensure uniform distribution of temperature throughout the vessel. Two Falcon test tubes were filled with phosphate-buffered saline containing 20% *v/v* of ethanol at pH values of 5.4 and 7.4 to represent the cancerous and healthy tissues’ media, respectively. The Falcon test tubes were then immersed into the beaker. Then, two dialysis bags were filled with nanoemulsions and placed inside the tubes. Samples were taken from the tubes 0, 0.5, 1, 2, 3, 6, 12, 24, 48, 72, and 96 h after the beginning of the experiment. The extracted sample was replaced with a proportional amount of fresh PBS. A UV-Vis spectrophotometer was used to quantify the amount of released quercetin within the samples. The percentage of released drug was calculated using Equation (3).

(3) $$Released\;Drug\;percentage=\frac{Released\;drug}{Loaded\;drug}\times 100$$

In Equation (3), “Loaded drug” is the amount of drug that was loaded in the nanoemulsions before placing them inside dialysis bags. “Released drug” is the amount of drug within the extracted samples, which was measured using a UV-Vis spectrophotometer. Since UV-Vis spectrophotometry was used to measure the amount of QC released from the different samples, the nanoparticles needed to have enough solubility in PBS. For this reason, 20% *v/v* ethanol was used to increase the solubility of the nanoparticles. The rationality of this method is consistent with previous literature reports on the dialysis bag technique [51,53,54].

### 2.9. Cell Culture

The A549 and L929 cell lines were cultured in RPMI 1640 and DMEM, respectively. The culturing procedure was performed with 100 µg/mL streptomycin, 100 U/mL penicillin, and 10% (*v*/*v*) fetal bovine serum. The cultivation media were kept in a humidified atmosphere containing 5% carbon dioxide.

### 2.10. MTT Assay

An MTT assay was employed to assess the cytotoxic effect of free QC, CMC, Fe_3_O_4_@SiO_2_, CMC/Fe_3_O_4_@SiO_2_, and CMC/Fe_3_O_4_@SiO_2_/QC on the A549 and L929 cell lines. Each well of a 96-well plate was filled with the cultivation medium, which contained 10^4^ cells. The plate was incubated for a day so that cell adhesion could happen. The cells were then treated with the abovementioned samples for 24 h. The reason for using various samples was to identify the effect of each component of the prepared nanocomposite on the antitumor activity of the whole system. In addition, the L929 cell line was also used to examine the cytotoxic effect of the prepared nanocarriers on a noncancerous cell line and obtain an estimate of the potential side effects of the delivery system. The control group was cultured in a similar medium (DMEM) without any treatment. After three days, the cells in each well were incubated with fresh DMEM and MTT solution. DMSO was also added to each well and stirred until formazan particles were dissolved. The control group was used as the reference for the reporting cell viability percentage of treated cells. An ELISA reader was used to determine optical density. All tests were performed in triplicate, and the standard error of the mean was calculated for each group using statistical data analysis methods.

### 2.11. Flow Cytometry Test

A flow cytometry test was employed to analyze the level of apoptosis and necrosis in A549 cells treated with CMC, Fe_3_O_4_@SiO_2_, CMC/Fe_3_O_4_@SiO_2_, and CMC/Fe_3_O_4_@SiO_2_/QC. After 24 h of treatment, the cells were washed with phosphate-buffered saline. This was followed by creating a suspension of cells in a binding buffer and staining them using Annexin V-FITC. A flow cytometer was used to measure apoptosis and necrosis by analyzing the fluorescence intensity. Four quadrants, for necrotic death (Q1), late apoptotic death (Q2), early apoptotic death (Q3), and viability (Q4), were defined, and quadrant statistics were performed. The testing of all samples was performed three times.

## 3. Results and Discussion

### 3.1. DLS

**Figure 1:**
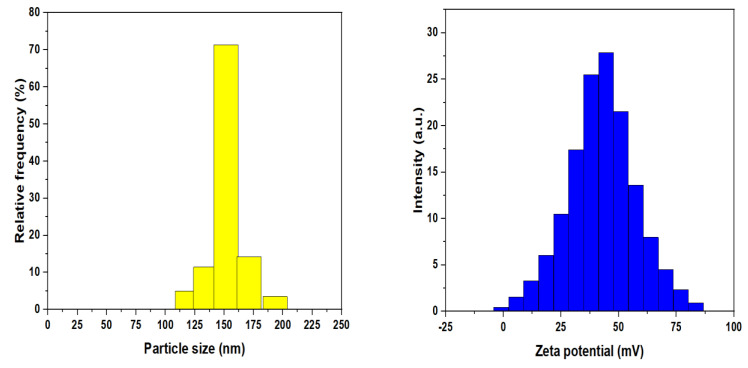
Particle size (**left**) and zeta potential (**right**) distributions for CMC/Fe_3_O_4_@SiO_2_/QC nanocomposite.

The dynamic light scattering (DLS) technique was used to determine the size of CMC/Fe_3_O_4_@SiO_2_/QC nanoparticles. The results indicated an average size of 151.6 nm of the nanoparticles, with a polydispersity index (PDI) of 0.14. It was evident that the obtained nanoparticles showed a uniform size distribution, since almost 70% of them had a size close to the average value. Moreover, the zeta potential values of the nanoparticles were measured to evaluate their stability. The average zeta potential value of the quercetin-loaded nanoparticles was 44.49 mV. According to literature reports, absolute zeta potential values higher than 30 mV are indicative of good colloidal stability [55]. Such high surface charge can prevent the aggregation of nanoparticles due to the repulsive electrostatic forces among them. Figure 1 shows the nanoparticles size distribution (left) and zeta potential distribution (right).

### 3.2. FE-SEM

**Figure 2:**
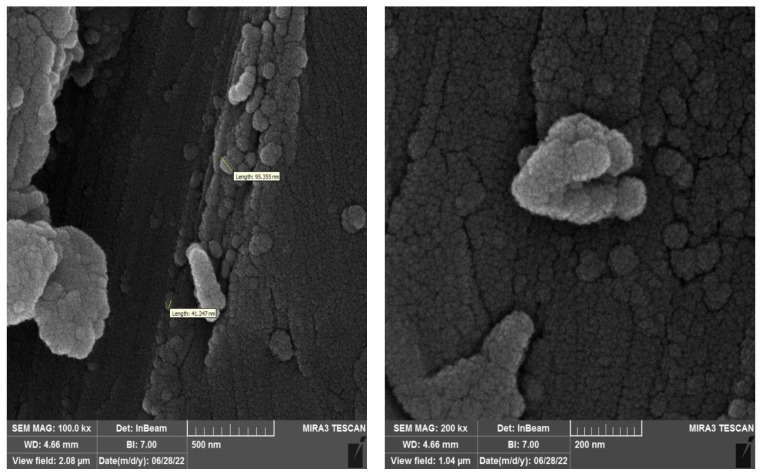
FE-SEM images of CMC/Fe_3_O_4_@SiO_2_/QC nanocomposites. Scale bar: 500 nm (**left**) and 200 nm (**right**).

The powder of CMC/Fe_3_O_4_@SiO_2_/QC was examined by means of FE-SEM to obtain insights into the morphology of the synthesized nanoparticles (Figure 2). It was evident from the images that the nanoparticles were spherical in shape, which is the most suitable geometry for drug delivery systems [56]. In addition, the nanoparticles displayed a smooth surface, which indicated good interphase adhesion between CMC and Fe_3_O_4_@SiO_2,_ likely attained via hydrogen bonding between the carboxyl groups of CMC and the hydroxyl groups of the inorganic nanoparticles, as evidenced in the FTIR analysis. It should be noted that the nanoparticles were dried using a freeze-dryer prior to SEM observation. As a result of this pretreatment, the particles were closely compacted together and appeared to show poor dispersity. Nevertheless, the DLS test, which measures the hydrodynamic size of the nanoparticles in solution, corroborated the uniform dispersity of the nanoparticles. As demonstrated in Figure 2, the nanoparticles had sizes well below 100 nm (e.g., 41.2 and 95.4 nm), which corroborates the nanoscale size of the delivery system. As expected, the obtained average size obtained with the DLS test was higher (around 150 nm), which was due to the agglomeration of some of the nanoparticles during the drying stage. A similar observation was made by Emami et al. [50] regarding the morphology of a chitosan/polyvinylpyrrolidone/α-Fe_2_O_3_ nanocomposite.

### 3.3. FTIR

**Figure 3:**
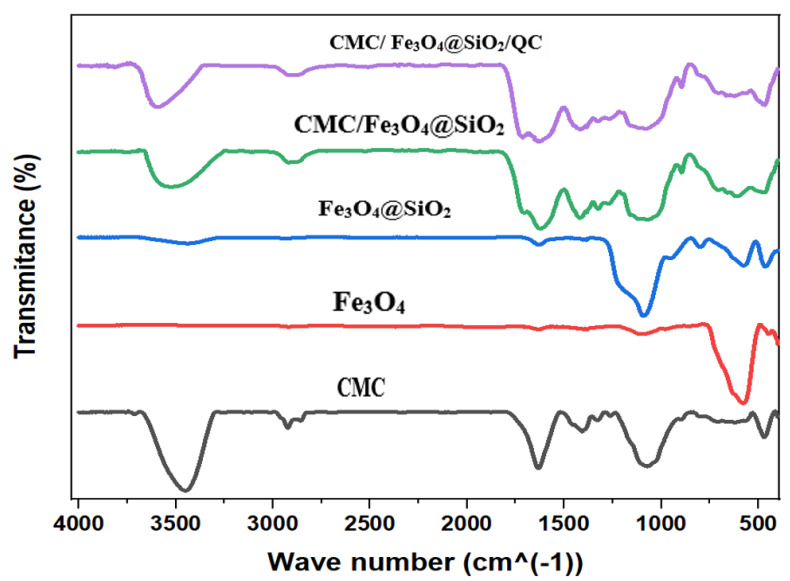
FTIR spectra of CMC, Fe_3_O_4_, Fe_3_O_4_@SiO_2_, CMC/Fe_3_O_4_@SiO_2_, and CMC/Fe_3_O_4_@SiO_2_/QC.

Fourier transform infrared spectroscopy was used to assess the chemical interactions among the functional groups of the different composite component bonds in the samples and to corroborate the sample composition. Figure 3 compares the FTIR spectra of CMC, Fe_3_O_4_, Fe_3_O_4_@SiO_2_, CMC/Fe_3_O_4_@SiO_2_, and CMC/Fe_3_O_4_@SiO_2_/QC. Regarding CMC, the peaks observed at 1407 and 1631 cm^−1^ were associated with symmetric and anti-symmetric stretching vibrations of the carboxyl group, respectively. Furthermore, the peak located around 3000 cm^−1^ indicated the stretching of the O-H bond. These results are in agreement with previous literature reports [21,57]. Regarding Fe_3_O_4_, the peak observed at 579 cm^−1^ indicated the stretching vibration of the metal–oxygen bond at the tetrahedral structure [58,59]. A new peak could be observed in Fe_3_O_4_@SiO_2_ at around 1100 cm^−1^, which could be assigned to the asymmetric vibration of bonds between oxygen and Si [60,61], which corroborated the formation of Fe_3_O_4_@SiO_2_ NPs. The FTIR spectrum of CMC/Fe_3_O_4_@SiO_2_ showed all the bands identified in both CMC and Fe_3_O_4_@SiO_2_. A decrease in the intensity of some of the peaks was observed, which was indicative of the interactions between inorganic core–shell nanoparticles and the polymeric network. Based on the results reported by Hu et al. [62], the peak observed at 1682 cm^−1^ could be attributed to hydrogen bonds between the Fe_3_O_4_@SiO_2_ nanoparticles and the carboxyl groups of CMC. Similarly, all of the characteristic peaks of CMC/Fe_3_O_4_@SiO_2_ were preserved once QC was loaded in the carrier. However, a shift in the peak located around 3500 cm^−1^ and the decreased intensity of some of the other peaks indicated interactions between quercetin and CMC. In addition, the peak observed at 1080 cm^−1^ could be associated with the vibration of the bond between Si and the OH group of quercetin [62].

### 3.4. XRD

**Figure 4:**
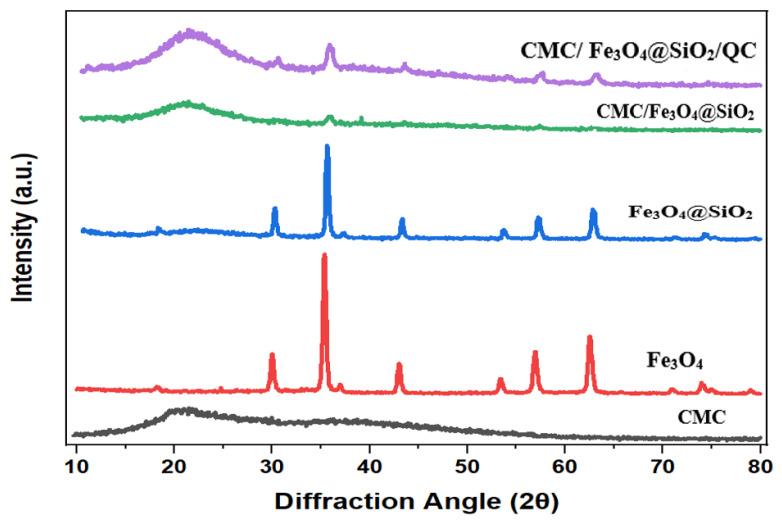
XRD patterns of CMC, Fe_3_O_4_, Fe_3_O_4_@SiO_2_, CMC/Fe_3_O_4_@SiO_2_, and CMC/Fe_3_O_4_@SiO_2_/QC.

The X-ray diffraction (XRD) technique was used to analyze the crystalline structures of CMC, Fe_3_O_4_, Fe_3_O_4_@SiO_2_, CMC/Fe_3_O_4_@SiO_2_, and CMC/Fe_3_O_4_@SiO_2_/QC. Figure 4 shows the XRD patterns of the abovementioned samples. The six peaks observed for Fe_3_O_4_ at 2ϴ angles of 30, 35.4, 43, 53.35, 56.9, and 62.55° were in very good agreement with previous data reported in the literature [63]. These results proved the successful synthesis of magnetite. Regarding CMC, the peak at a diffraction angle of 21.84° was also consistent with former studies [64]. Regarding Fe_3_O_4_@SiO_2_ nanoparticles, the XRD pattern depicted six peaks at diffraction angles of 30.34, 35.74, 43.34, 53.74, 57.24, and 62.89°, which were in agreement with a former XRD analysis of these nanoparticles [65]. The broad peak of CMC at 2θ = 21.84° indicated its amorphous structure. The XRD pattern of CMC/Fe_3_O_4_@SiO_2_ was quite similar to that of CMC. However, a peak was observed at 2θ = 35.4°, which confirmed the successful incorporation of Fe_3_O_4_@SiO_2_ nanoparticles within the CMC structure. The decreased intensity of this peak could be associated with the amorphous structure of CMC. Furthermore, it could be seen from the XRD results that no noticeable peaks were added to the pattern of CMC/Fe_3_O_4_@SiO_2_/QC compared with that of unloaded nanocomposites. This result corroborated the entrapment of quercetin within the nanocomposite network. The encapsulation of quercetin within the nanocomposites inhibited crystal formation, which is a desirable result, as the crystalline form of quercetin is less soluble than its amorphous form [54].

### 3.5. VSM

**Figure 5:**
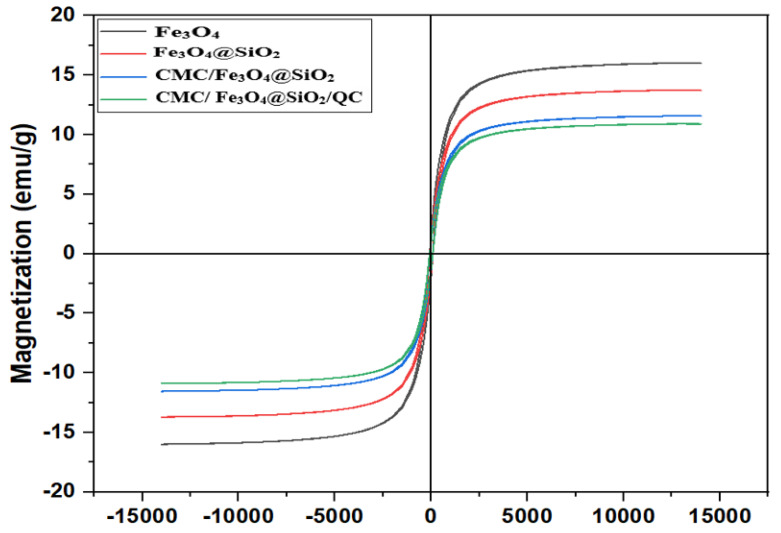
VMS results for Fe_3_O_4_, Fe_3_O_4_@SiO_2_, CMC/Fe_3_O_4_@SiO_2_, and CMC/Fe_3_O_4_@SiO_2_/QC.

A vibrating sample magnetometer (VSM) was employed to determine hysteresis loops in Fe_3_O_4_, Fe_3_O_4_@SiO_2_, CMC/Fe_3_O_4_@SiO_2_, and CMC/Fe_3_O_4_@SiO_2_/QC (Figure 5). The strength of the applied magnetic field was in the range of −15,000 to +15,000 kilo oersted. The curves of all samples were S-shaped and had zero coercivity, which revealed their super magnetic characteristic. Furthermore, it can be observed from Figure 5 that upon the addition of each component (SiO_2_, CMC, and QC), the saturation magnetization value decreased. For instance, while this value was approximately −16 emu/g for Fe_3_O_4_, it fell to around −11 emu/g for CMC/Fe_3_O_4_@SiO_2_/QC. This behavior could be attributed to the drop in the weight percentage of Fe_3_O_4_ upon the incorporation of the non-magnetic components.

### 3.6. Loading and Entrapment Efficiency

As mentioned before, a major challenge that inhibits the therapeutic effectiveness of QC is its low solubility, which leads to insufficient bioavailability [66]. In light of this fact, finding ways to improve the loading efficiency and entrapment efficiency of the drug on the developed nanocarriers is essential. Herein, we measured the loading efficiency and entrapment efficiency of the drug on both the CMC and CMC/Fe_3_O_4_@SiO_2_ hydrogels to evaluate the impact of incorporating the core–shell nanoparticles in the polymeric structure. The loading efficiency and entrapment efficiency were calculated using Equations (1) and (2), respectively. Upon the incorporation of Fe_3_O_4_@SiO_2_ NPs within the hydrogel network, the loading efficiency increased from 86.75% to 88.50%. In addition, the entrapment efficacy had a 2% rise from 45.00% to 47.25%. These data are summarized in Table 1. Based on the FTIR spectrum of the final formulation (CMC/Fe_3_O_4_@SiO_2_/QC), the peak around 1080 cm^−1^ could be attributed to the vibration of the bond between Si and the hydroxyl group of quercetin. This characteristic could explain the slight improvement in the loading of quercetin and suggests that quercetin was mostly loaded in the SiO_2_ particles. A similar result derived from an FTIR analysis was reported by Hu et al. [62].

Although the loading and entrapment efficiency did not improve significantly after introducing the core–shell nanoparticles, the FTIR analysis revealed that in the presence of these nanoparticles, a share of loaded quercetin is entrapped inside them instead of the polymeric network. This entrapment contributes to the sustained release of quercetin, as the drug has to diffuse through extra layers before getting released. In the following sections, it is shown that this sustained release profile improves anticancer efficiency of the drug delivery system.

### 3.7. Quercetin Release Profile

**Figure 6:**
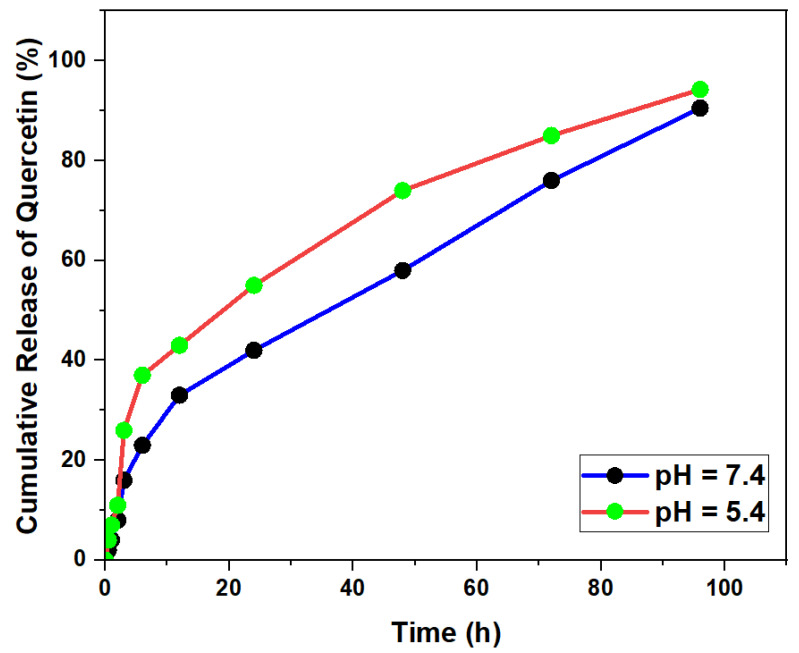
Cumulative curves of quercetin release from the nanocomposite carriers.

The abovementioned dialysis method was employed to study the in vitro release of quercetin from the CMC/Fe_3_O_4_@SiO_2_ nanocomposite. Figure 6 shows the cumulative release curve of quercetin throughout 96 h. The release profile of the drug was studied at pH values of 7.4 and 5.4. Buffer solutions with the mentioned pH values were used to simulate the microenvironment of normal and cancerous tissues, respectively. Both solutions were kept at 37 °C, which is the physiological temperature. The solutions were monitored for 96 h. Within the first 12 h, the cumulative amounts of the drug released from nanocarriers within neutral and acidic media were 33 and 43%, respectively. Wang et al. [67] examined the release profile of QC from F127, used as the carrier. Within 12 h, approximately 70% of quercetin was released in the neutral environment. Baksi and coworkers [68] studied release of quercetin from chitosan nanoparticles and obtained around 60% cumulative release within 12 h. In another study, Sunoqrot and colleagues [69] obtained about 60% release of quercetin from Eudragit S100 nanoparticles within 12 h at a pH of 7.2. Compared with the mentioned literature reports, the nanocomposite developed herein releases quercetin in a more sustained manner. This sustained release pattern in neutral environment is of paramount importance, since the administered nanoparticles have to pass through several normal tissues before reaching the tumor microenvironment. Hence, it is beneficial for the nanocarrier to retain most of the payload before reaching tumor sites. In light of this explanation, the sustained release pattern of quercetin can minimize the side effects of the drug. Likely, the stability of the quercetin molecules inside the nanocomposite network that makes this sustained release pattern feasible can be associated with the formation of hydrogen bonds between the hydroxyl groups of quercetin and the carboxylic acid groups of CMC [40].

Furthermore, the difference in the quercetin release profile under acidic and neutral conditions is an evident indicator of the pH sensitivity of the CMC/Fe_3_O_4_@SiO_2_ nanocomposite. Within 24 h, 42 and 58% of quercetin were released at pH values of 7.4 and 5.4, respectively. The increased amount of drug released under acidic pH conditions can be associated with the protonation of the carboxylic acid groups, which leads to the disruption of the hydrogen bonds between hydroxyl and carboxyl groups [62]. The pH sensitivity of the nanoparticles is a key factor in improving their therapeutic efficiency and decreasing the side effects. Many tissues of body and the blood itself have neutral pH values (close to 7.0), whereas the microenvironment of tumors has acidic pH. Provided that our nanoparticles are administered via intravenous injection, they would bypass the GI tract and reach systemic blood circulation directly. Hence, they would retain most of their therapeutic payload until they reach the tumor site, where the drug would be released. In this way, healthy tissues would be preserved from the toxicity of quercetin drug. In addition, the benefits of avoiding the GI tract include preventing the dissociation of the nanoparticle structure in the highly acidic environment of the stomach (pH < 2.0). Many literature reports have focused on developing similar pH-sensitive delivery systems for cancer therapy [70,71,72,73,74,75,76,77,78,79].

The incorporation of the nanocomposites within water-in-oil-in-water double emulsion also played a role in controlling the release of quercetin. The oil layer of the double-emulsion system acted as a membrane and inhibited the high burst release of quercetin after the disintegration of the nanocomposite network. In addition, the presence of SPAN 80 as a surfactant contributed to stabilize the nanoemulsions and to extend the drug release. Pourmadadi et al. [80] incorporated 5-fluorouacil/curcumin-loaded nanocomposites of agarose/chitosan within double nanoemulsions of W-O-W and obtained similar results in terms of release profile.

### 3.8. Drug Release Kinetic Modelling

**Figure 7:**
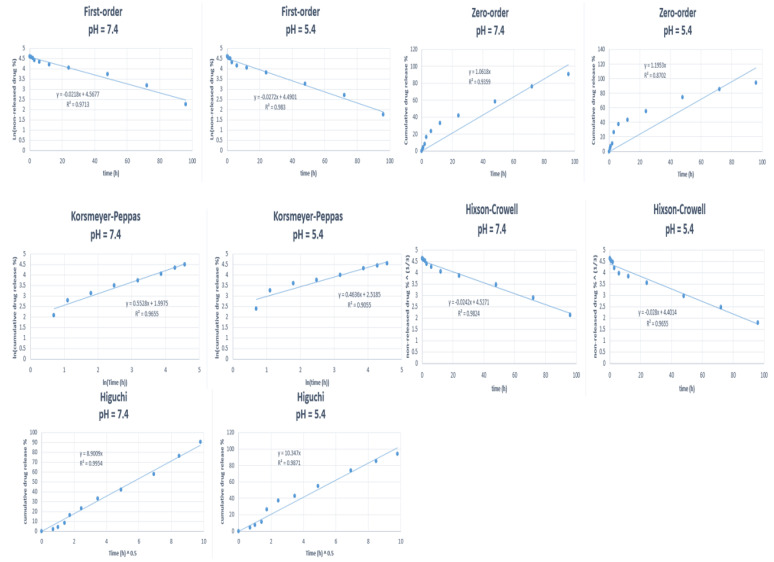
Fitting of the drug release data to different kinetic models.

The data obtained with in vitro drug release studies were used to develop a kinetic model for quercetin release. The data for both pH values of 7.4 and 5.4 were fitted to various models, including the first-order model, the zero-order model, the Korsmeyer–Peppas model, the Hixson–Crowell model, and the Higuchi model. By comparing the R-squared values of the different models, the Higuchi model was identified as the most accurate one for describing the profile of quercetin release data. Furthermore, hypothesis testing was performed to analyze the significance of the time variable in each model. All models had *p*-values lower than 0.0001, which indicated the significance of the chosen variable for modelling. Assuming that the null hypothesis claims the insignificance of the time variable on drug release, *p*-values lower than 0.0001 were proof for rejecting this hypothesis and validated the models. Figure 7 shows the fitting of the release data to different models.

The Higuchi model was the first mathematical model applied to study drug release from a porous media such as a porous polymer. Higuchi divides the porous medium in two regions. The first one is the inner region, which contains undissolved particles. The second one is the outer region, where drug particles are dissolved. The assumptions that need to be upheld upon using this model and that are thus valid in our system include the following: (i) drug diffusivity does not change; (ii) the size of the drug particles is negligible compared with the thickness of the medium walls; (iii) The drug diffuses in one dimension only; (iv) the initial drug concentration in the porous media is greater than its solubility; (v) the swelling and disintegration of the porous medium can be neglected. The formula of this model can be written as $Q=A\sqrt{D\left(2C-C_{s}\right)C_{s}t}$. In this formula, *Q* is the amount of released drug, *t* is the time, *A* is the area, *C* is the initial drug concentration, and $C_{s}$ is the drug solubility in the porous media [81,82]. Table 2 shows the equations and R-squared values of the different kinetic models applied to quercetin release in both acidic and neutral environments.

### 3.9. MTT Assay

**Figure 8:**
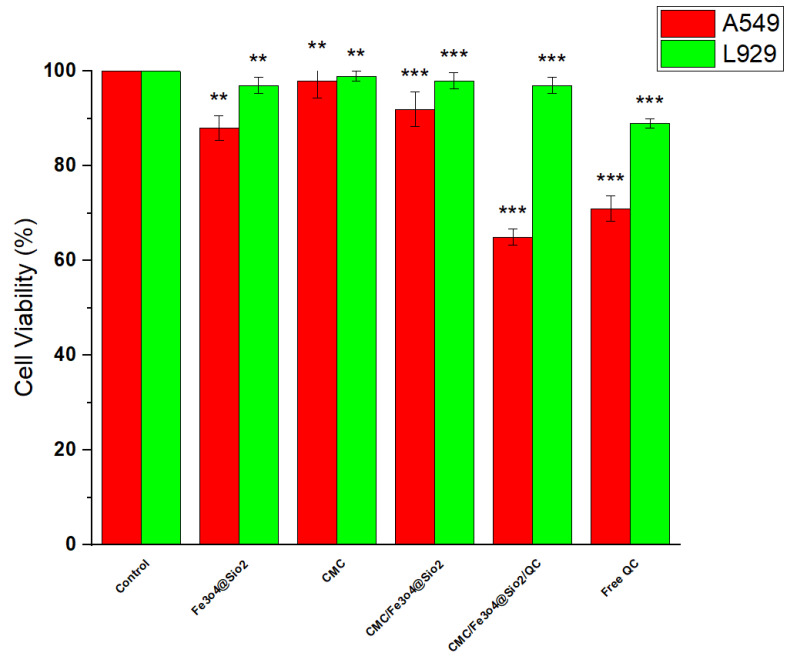
MTT assay results in a 24 h time frame. Incubation was performed in a 96-well plate, and the experiments were performed three times for each sample. The data are shown as means ± SEMs. (Difference between control group and the samples marked with ** was significant at *p* < 0.01; difference between control group and the samples marked with *** was significant at *p* < 0.001.)

An MTT assay was performed to assess the in vitro cytotoxicity of the prepared nanocarriers against the A549 lung cancer cell line and compare its antitumor activity with that of free quercetin. The same experiments were performed on the L929 fibroblast cell line to evaluate the potential side effects of the prepared nanocarriers. Both cell lines were incubated with Fe_3_O_4_@SiO_2_, CMC, CMC/Fe_3_O_4_@SiO_2_, CMC/Fe_3_O_4_@SiO_2_/QC, and free quercetin. The concentration of each sample in each experiment was 5 µg/mL. A positive control group was also cultured to verify the cells’ natural growth and proliferation. All experiments were performed three times to validate the accuracy of the results. The data in Figure 8 are reported as means ± SEMs.

The control group had 100% cell viability after 24 h, which corroborated the quality of the selected cells. Other than the control group, all the samples used in different experiments, including the final CMC/Fe_3_O_4_@SiO_2_/QC nanocomposite, induced higher cytotoxicity in A549 cells compared with L929 cells. While CMC did not have any noticeable toxic impact on either of the cell lines (similar to the results presented in previous literature reports on CMC biological properties [20,83]), Fe_3_O_4_@SiO_2_ nanoparticles eliminated around 15% of cancerous cells and exhibited the cytotoxic property. Shahabadi et al. [84] reported a similar biological behavior in Fe_3_O_4_@SiO_2_ nanoparticles. However, their impact on the viability of L929 cells was still negligible, which is a desirable result. As expected, encapsulating these inorganic nanoparticles within the CMC hydrogel mitigated their cytotoxic effects on cancerous cells. Loading quercetin on the CMC/Fe_3_O_4_@SiO_2_ hydrogel decreased the viability percentage of A549 cells to 65%, which was 27% less than the value of the raw nanocomposites. In addition, 97% of L929 cells survived 24 h of incubation with CMC/Fe_3_O_4_@SiO_2_/QC nanocomposites. The viability percentages of A549 and L929 cells after 24 h of incubation with free quercetin were 71 and 89%, respectively. Sul and colleagues [85] performed an MTT assay using the A549 cell line and different concentrations of free quercetin. The lowest viability percentages of the cells hardly reached 70%, and the results were in agreement with this work. Nanomaterials loaded with Quercetin as an advanced tool for cancer treatment were reviewed by Caro and coworkers [86]. In addition, Milanezi et al. [87] performed an MTT assay using free quercetin and the L929 cell line and obtained cell viability values similar to those of this study. The selectivity of quercetin between normal cells and cancerous cells has been reported in the literature [88]. The comparison of these values with their counterparts in the CMC/Fe_3_O_4_@SiO_2_/QC experiment is a clear indication of the superiority of the synthesized nanocomposites in terms of cytotoxicity and side effects. Quercetin-loaded nanocomposites eliminated more cancerous cells, whereas their effect on L929 fibroblast cells was less than that of the free drug. The entrapment of quercetin within core–shell nanoparticles and their consequent sustained release from the double-layer nanoemulsion led to the boosted apoptosis of A549 cells. This sustained release pattern of quercetin is of paramount importance, since this drug has low bioavailability and its sustained release can lead to prolonged exposure of cancerous cells to it.

It could be observed that incubating A549 cells with CMC/Fe_3_O_4_@SiO_2_/QC nanoparticles did not offer a very low viability percentage (65%). This result can be attributed to the low concentration of the nanoparticles in the experiments (5 µg/mL). Here, we demonstrated that strategies such as using core–shell nanoparticles and a double-emulsion system could lead to a sustained release profile of the loaded drug, thereby decreasing the cell viability compared with the free quercetin. It is expected that increasing the initial dosage of the nanoparticles results in the elimination of more cancerous cells. It is worth mentioning that the potential low anticancer potency of quercetin could not have been responsible for this result, as multiple studies have already reported its efficient anticancer activity against various cell lines [3,51].

### 3.10. Flow Cytometry Test

**Figure 9:**
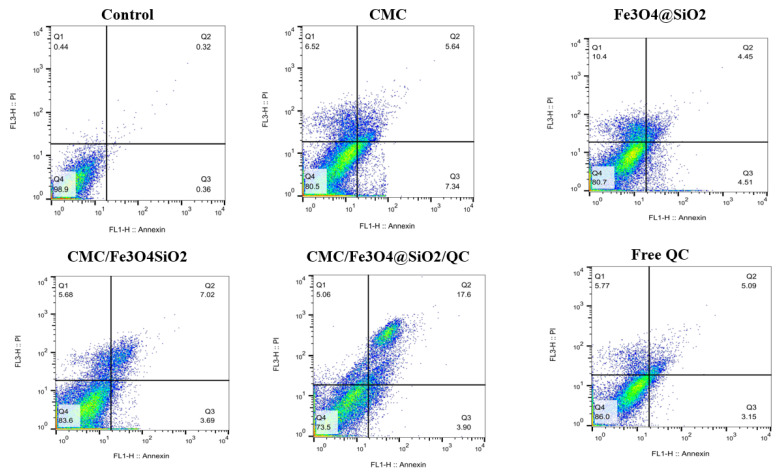
Results of flow cytometry test for CMC, Fe_3_O_4_@SiO_2_, CMC/Fe_3_O_4_@SiO_2_, CMC/Fe_3_O_4_@SiO_2_/QC, and free QC using the A549 cell line.

In order to analyze the cytotoxic impact of the designed system in more detail and verify the results obtained in the MTT test, a flow cytometry test was performed with free QC, CMC, Fe_3_O_4_@SiO_2_, CMC/Fe_3_O_4_@SiO_2_, and CMC/Fe_3_O_4_@SiO_2_/QC using the A549 cell line. The concentration of each sample for each experiment was 5 µg/mL. Figure 9 demonstrates the results of flow cytometry for each sample. As mentioned before, Q1, Q2, Q3, and Q4 quadrants represent necrotic death, late apoptotic death, early apoptotic death, and variability, respectively. It can be interpreted from Figure 9 that both CMC and Fe_3_O_4_@SiO_2_ NPs had lower cell viability percentages than the nanocomposite form of the components (CMC/Fe_3_O_4_@SiO_2_). Hence, the fabricated hydrogel that contained Fe_3_O_4_@SiO_2_ NPs ameliorated the toxicity of both the polymeric component and the core–shell NPs. Nevertheless, the final formulation of the raw nanocarrier was noticeably cytotoxic itself, as 5.68%, 7.02%, and 3.69% of incubated cells were subjected to necrotic, late apoptotic, and early apoptotic death, respectively. Upon the encapsulation of QC within the nanocarrier network, a noticeable increase was observed in the apoptosis percentage with the increase in the share of cancerous cells subjected to either early or late apoptosis from 10.71% to 21.50% compared with the raw nanocarriers. The comparison of the cell viability values of the control group, the raw nanocarriers, and the loaded nanocarriers was consistent with the results obtained from MTT assay. Moreover, the QC-loaded nanoemulsion demonstrated superior performance in terms of apoptosis induction compared with free QC. While loaded nanoemulsions eliminated 21.50% of A549 cells through either early or late apoptosis, this figure, for free QC, was no more than 8.24%. This result was also in agreement with the MTT assay data. The significant difference between the apoptosis values of QC-loaded nanocarriers and free QC was likely associated with the very high value of late apoptosis (Q2) of the loaded nanocarriers. The strong interactions between entrapped QC and the polymeric and inorganic components of the nanocomposite, together with the encapsulation of the nanocomposites within the double-layer nanoemulsion, led to a very gradual release pattern. Such sustained release profile and the hindrance of burst release led to greater late apoptotic-mediated cell death. The share of the cells subjected to necrotic death hardly changed from the raw nanoemulsion to the loaded nanoemulsion or the free drug.

## 4. Conclusions

Drawbacks such as ineffective biodistribution, insufficient biological half-life, low solubility, and instability have limited the therapeutic efficiency of quercetin drug against cancer. In this regard, the design of novel stimulus-responsive drug delivery systems that can extend the release time of QC, improve its loading efficiency, and trigger its release at specific target sites is necessary to circumvent these disadvantages. pH is an important factor that can markedly affect oral drug absorption and bioavailability, as the pH values of normal tissues and tumors are noticeably different. In this regard, pH-responsive nanocarriers can be developed for effective cancer therapy. In this study, a pH-responsive nanocomposite was synthesized based on CMC and Fe_3_O_4_@SiO_2_ core–shell nanoparticles to improve the controlled release of QC at tumor sites. In addition, the nanocomposites were entrapped in a double-layer nanoemulsion to further extend the QC release time. In vitro drug release studies revealed that the developed nanoemulsion retained most of the payload in neutral medium and released a higher amount of drug in acidic environment due to the protonation of the carboxyl groups and the dissociation of hydrogen bonds. The prolonged drug release period and the pH-sensitiveness can account for the low bioavailability and inefficient biodistribution of QC, respectively. In addition, the incorporation of inorganic core–shell NPs with high surface area within the polymeric network slightly improved the loading efficiency of QC. Furthermore, an MTT assay and a flow cytometry test using the A549 cell line demonstrated the higher effectiveness of the QC-loaded nanoemulsion compared with free QC in killing cancerous cells and inducing apoptosis. An MTT assay was also performed using the L929 cell line, and the results demonstrated a low cytotoxic effect of the nanoemulsion on the fibroblast cell line compared with the free drug, which can be an indicator of low side effects of the developed nanocarrier. To sum up, a novel pH-responsive double-emulsion-entrapped hydrogel nanocomposite was prepared for effective cancer therapy.

## Figures and Tables

**Table 1:** Entrapment and loading efficiency of CMC and CMC/Fe_3_O_4_@SiO_2_ hydrogels.

Hydrogel	Entrapment Efficiency (%)	Loading Efficiency (%)
CMC	45.00	86.75
CMC/Fe_3_O_4_@SiO_2_	47.25	88.50

**Table 2:** Equations and R-squared values for different kinetic models applied to drug release data.

Model	Equation	R^2^
First-order (pH = 7.4)	$Ln\left(1-\frac{M_{t}}{M_{\infty }}\right)=4.5677-0.0218t$	0.9713
First-order (pH = 5.4)	$Ln\left(1-\frac{M_{t}}{M_{\infty }}\right)=4.4901-0.0272t$	0.9830
Zero-order (pH = 7.4)	$C_{t}=1.0618t$	0.9359
Zero-order (pH = 5.4)	$C_{t}=1.1953t$	0.8702
Korsmeyer–Peppas (pH = 7.4)	$Ln\left(\frac{M_{t}}{M_{\infty }}\right)=1.9975+0.5528Ln\left(t\right)$	0.9655
Korsmeyer–Peppas (pH = 5.4)	$Ln\left(\frac{M_{t}}{M_{\infty }}\right)=2.5185+0.4636Ln\left(t\right)$	0.9055
Hixson–Crowell (pH = 7.4)	${\left(1-\frac{M_{t}}{M_{\infty }}\right)}^{\frac{1}{3}}=4.5271-0.0242t$	0.9824
Hixson–Crowell (pH = 5.4)	${\left(1-\frac{M_{t}}{M_{\infty }}\right)}^{\frac{1}{3}}=4.4014-0.028t$	0.9655
Higuchi (pH = 7.4)	$Q=8.9009t^{0.5}$	0.9954
Higuchi (pH = 5.4)	$Q=10.347t^{0.5}$	0.9871

## Data Availability

Data within this article are available upon request.
